# CIRCADIAN CLOCK-ASSOCIATED 1 Inhibits Leaf Senescence in *Arabidopsis*

**DOI:** 10.3389/fpls.2018.00280

**Published:** 2018-03-06

**Authors:** Yi Song, Yupei Jiang, Benke Kuai, Lin Li

**Affiliations:** State Key Laboratory of Genetic Engineering and Institute of Plant Biology, School of Life Sciences, Fudan University, Shanghai, China

**Keywords:** circadian clock, leaf senescence, CCA1, ORE1, GLK2, aging

## Abstract

Leaf senescence is an integral part of plant development, and the timing and progressing rate of senescence could substantially affect the yield and quality of crops. It has been known that a circadian rhythm synchronized with external environmental cues is critical for the optimal coordination of various physiological and metabolic processes. However, the reciprocal interactions between the circadian clock and leaf senescence in plants remain unknown. Here, through measuring the physiological and molecular senescence related markers of several circadian components mutants, we found that CIRCADIAN CLOCK-ASSOCIATED 1 inhibits leaf senescence. Further molecular and genetic studies revealed that CCA1 directly activates *GLK2* and suppresses *ORE1* expression to counteract leaf senescence. As plants age, the expression and periodic amplitude of *CCA1* declines and thus weakens the inhibition of senescence. Our findings reveal an age-dependent circadian clock component of the process of leaf senescence.

## Introduction

Leaf senescence is an integral part of plant development coupled with dramatic transcriptome reprogramming and the massive breakdown and recycling of macromolecules (Lim et al., 2007; Breeze et al., 2011; Wu et al., 2012). Senescence occurs autonomously in an age-dependent manner controlled by an innate genetic program and environmental stresses. Molecular genetic studies using *Arabidopsis thaliana* have identified many senescence-associated genes (SAGs) (leaf senescence database^1^) (Li et al., 2014). Among them, *ORESARA 1* (*ORE1*, also named *NAC2*, *NAC6*, and *NAC092*) is an age- and ethylene-induced senescence regulator that positively regulates senescence initiation and chlorophyll degradation (Kim et al., 2009; Qiu et al., 2015). GLKs (Golden2-like transcription factors) act as critical maintainers of chloroplast activity and overexpressing *GLK2* delays leaf senescence (Waters et al., 2009). A recent study showed that the protein interaction between ORE1 and GLKs (GLK1, GLK2) regulates the balance between growth and senescence. The progressive accumulation of ORE1 counteracts the function of GLKs and eventually shifts the balance toward senescence initiation (Rauf et al., 2013).

Circadian clocks orchestrate diverse developmental and physiological processes to enhance adaptability to diurnally changing environmental cues (Harmer, 2009). Loss of circadian regulation dampens fitness (Green et al., 2002). CIRCADIAN CLOCK ASSOCIATED 1 (CCA1) and its homologous protein LATE ELONGATED HYPOCOTYL (LHY), together with TIMING OF CAB EXPRESSION 1 (TOC1), form a core loop by repressing one another’s expression (Nagel and Kay, 2012). Circadian output regulates diverse processes such as photosynthesis, chlorophyll and starch metabolism (Harmer, 2000), hormone synthesis and signaling (Zheng et al., 2015) and the stress response (Wang et al., 2011; Takeuchi et al., 2014) to modulate their activity differentially at different time points of the day, which is known as the “gating” effect.

There are increasing lines of evidence that disruptions to the circadian system can substantially impact aging in animals (Banks et al., 2016), but the reciprocal interaction between circadian clock and senescence in plant systems has not been studied. Here, we investigated leaf senescence process in circadian clock-related mutants and revealed the possible mechanisms of inhibition of CCA1 in leaf senescence. With aging, the reduced expression of *CCA1* attenuates the inhibition and leads to accelerated leaf senescence.

## Materials and Methods

### Plant Material and Growth Conditions

*Arabidopsis thaliana* ecotype Col-0 was used as the wild type in this study. The mutants used have been described previously: *cca1-1* (Green and Tobin, 1999) (Ws background back-crossed with Col-0 three times), *cca1-11* (Hall et al., 2003) (Ws background back-crossed with Col-0 three times), *lhy* (Salk_031092), *cca1lhy*, *elf3-7* (Hicks et al., 2001), and *toc1-2* (Strayer et al., 2000) (C24 background back-crossed with Col-0 three times). The mutant line *ore1* (SALK_090154) was obtained from ABRC, and *cca1-1ore1* was generated by crossing. The CDS (coding sequence) of *GLK2* was cloned into pCMBIA1302 and then transformed into *Agrobacterium* strain GV3101 to generate line 35S:GLK2/*cca1-1*. Seeds were cold-treated at 4°C for 3 days. Seedlings were grown in soil under 100 μmol m^-2^ s^-1^ fluorescent light in a 22°C growth room with relative humidity of 65%.

### Electrophoretic Mobility Shift Assay

The full-length *CCA1* CDS was cloned into pMAL-c5g, and the empty vector pMAL-c5g was used for MBP expression. Plasmids were transformed into *Escherichia coli* strain Rosetta (DE3), and the proteins were induced by 0.5 mM isopropyl thio-β-D-galactoside (IPTG) at 18°C for 16 h in 200 mL LB medium. Amylose resin (New England Biolabs, United States) was used to purify MBP-CCA1 fusion protein and MBP following the manufacturer’s instructions. EMSA was conducted as described previously (Qiu et al., 2015).

### Chromatin Immunoprecipitation

Detached leaves from 4-week-old Col-0 and CCA1OX (*35S:CCA1-Flag*) plants (Li et al., 2011) were collected at ZT1. Flag M2 antibody (Sigma) was used for immunoprecipitation. ChIP-qPCR results were first normalized with input sample as follows: cycle threshold (Ct) = Ct_ChIP_-Ct_Input_. Relative enrichment was then calculated as the ratio of normalized results from *35S:CCA1-Flag* relative to the WT control. Primers are listed in Supplementary Table 2.

### Dual-Luciferase Transient Expression Assay

The promoters of *ORE1* (2043 bp) and *GLK2* (1589 bp) were amplified from Col-0 genomic DNA and cloned into the transient expression vector pGreenII0800-Luc to generate reporter constructs. The full-length *CCA1* CDS was cloned into the pCHF3 binary vector to generate an effector construct. The constructs were transformed into *Agrobacterium* strain GV3101. A mixture of cells containing constructs with both effector and reporter and pSoup-P19 was infiltrated into young *Nicotiana benthamiana* leaves. The firefly and Renilla luciferase activities of *Nicotiana benthamiana* extracts were quantified using the Dual-Luciferase Reporter Assay System (Promega, United States) and detected with a Synergy 2 multi-mode microplate (Bio-Tek).

### Physiological Measurements

Relative chlorophyll contents were measured using a Konica Minolta SPAD 502 dual-wavelength chlorophyll meter; we measured three leaf areas from the tip to base, and the average value was used for further analysis. Measurements of photochemical efficiency (Fv/Fm) were conducted as described previously (Song et al., 2014). DAB staining was conducted as in a previous report (Lai et al., 2012). Ethylene treatment was conducted as described previously (Liu and Wen, 2012).

### Transcript Analysis

Total RNA of leaves was extracted using an RNAiso plus kit (Takara). A Reverse Transcription System (Takara) was used for first-strand cDNA synthesis. Quantitative PCR was performed with SYBR^®^ premix Ex Taq^TM^ II (Takara) on the MyiQ2 Real Time PCR Detection System (Bio-Rad, Hercules, CA, United States), and *ACTIN2* was used as an internal control to normalize the gene expression level. Primers used in this study are listed in Supplementary Table 2.

## Results

### CCA1 Negatively Regulates Leaf Senescence

**FIGURE 1:**
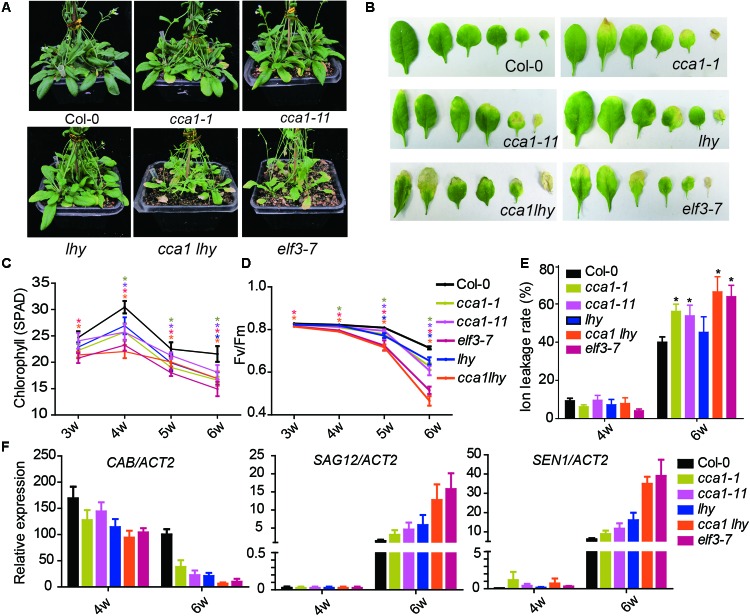
Circadian related mutants show altered physiological parameters of senescence. **(A)** Phenotypes of the Col-0 and circadian related mutants at 6 weeks under long day after germination. **(B)** Representative rosette leaves from different genotypes at 6 weeks under long day after germination. Chlorophyll contents (measured as SPAD) **(C)**, Fv/Fm ratios **(D)** and ion leakage rates **(E)** of the 5th and 6th true leaves from different genotypes measured at 3, 4, 5, and 6 weeks after germination. Asterisks indicate where the difference between Col-0 and mutant is statistically significant (^∗^*P* < 0.05). **(F)** Relative expressions of *CAB*, *SAG12*, and *SEN1* were detected in the 5th and 6th true leaves from 4- and 6-week-old long day grown plants. Three biological replicates were performed. *ACTIN2* is an internal control for quantitative real-time PCR.

To investigate the effect of the circadian clock on leaf senescence, we examined the physiological parameters of senescence in several circadian clock component mutants (*cca1-1*, *cca1-11*, *lhy*, *cca1lhy*, and *elf3-7*) (**Figure 1**). After 6 weeks’ long day growth (16 h light and 8 h dark, 16L/8D), all detected circadian mutants displayed faster progression than Col-0 of senescence related indexes. The *cca1-1*, *cca1-11*, and *lhy* single mutants exhibited a significantly accelerated yellowing phenotype relative to Col-0, and the *cca1lhy* double mutant aggravated the early senescence phenotype. *elf3-7* displayed a similar phenotype with that of *cca1lhy* (**Figure 1A**). Typical rosette leaves of 6-week old plants from the different genotypes showed the leaf longevity to evident the senescence process (**Figure 1B**). The chlorophyll content, Fv/Fm and ion leakage of the 5th and 6th true leaves was recorded from the third week to the sixth week after germination (**Figures 1C–E**). The single mutants of *cca1* and *lhy* seemed normal at the third week but initiated accelerated changes on all the measured physiological parameters from the fourth week. The mutant of *cca1lhy* and *elf3-7* had reduced chlorophyll content and Fv/Fm at the start of measurements. It has been known that single mutant of *cca1-1* and *lhy* flowers at a similar time to wild type under long day, but earlier than wild type under short day (Mizoguchi et al., 2002). When photoperiod was changed to 12L/12D (12 h light and 12 h dark) growth condition, early senescence process in *cca1-1* and *lhy* was even exaggerated (Supplementary Figure 1). Moreover, the changes of transcriptional level of senescence marker genes (*CAB*, *SAG12*, and *SEN1*) were also enhanced in circadian mutants after 6 weeks long day growth (**Figure 1F**). Taken together, these phenotypic and gene expression data suggest that circadian modulators, such as CCA1, LHY and ELF3, are negative regulators of natural leaf senescence.

Actually, it is not surprising of the earlier senescence in these circadian mutants because some of them show earlier flowering phenotypes (Harmer and Kay, 2005). Although the molecular link between flowering and senescence is not built yet and *cca1-1* displayed the similar flowering time with wild type under long day, the same chronological age but different physiological age/stage may confuse the consequence of phenotypes. Plant hormones, such as abscisic acid (ABA) and jasmonic acid, can rapidly induce detached leaf senescence (Zhu et al., 2015; Gao et al., 2016). To further verify the involvement of CCA1 in senescence, we investigated ABA and MeJA-induced leaf senescence in detached leaf of *cca1-1*. Before the treatment, the physiological stage (Chlorophyll contents and Fv/Fm) of leaves from Col-0 and *cca1-1* is similar. After 3 days ABA and MeJA treatments, accelerated senescence was observed in *cca1-1* compared with that in Col-0 (Supplementary Figure 2). CCA1 might play a part in these hormone responses, but CCA1 is involved in the regulation of leaf senescence process.

### Bioinformatics Screen for Targets of CCA1

The negative regulations of CCA1 on leaf senescence could be caused by (1) the overall physiological state due to shorter period (Dodd et al., 2005; Ni et al., 2009; Graf et al., 2010; Nagel and Kay, 2012); (2) the role on hormone responses (Mizuno and Yamashino, 2008; Atamian and Harmer, 2016); (3) the suppression effect of CCA1 on senescence associated genes (SAGs), and so on. Among these possibilities, we investigated the direct regulation of CCA1 on SAGs firstly.

**FIGURE 2:**
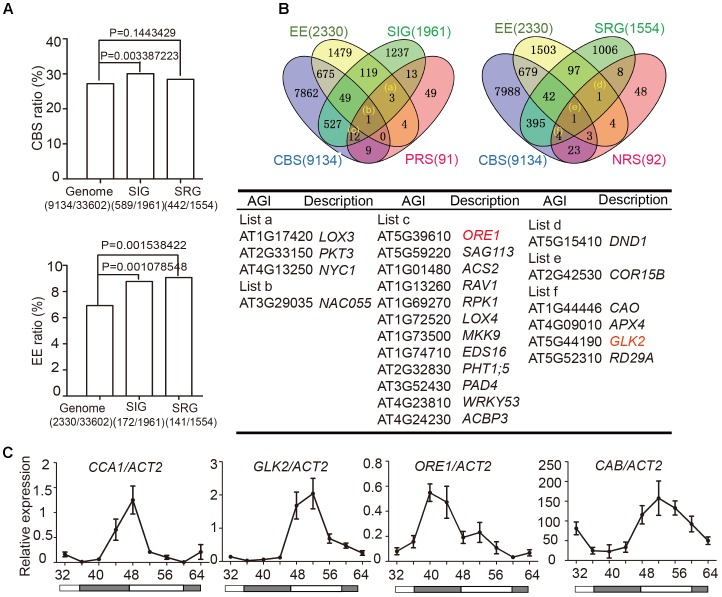
Identification of circadian-associated senescence regulators. **(A)** The ratios of CBS and EE in genome background, senescence induced genes (SIGs) and senescence repressed genes (SRGs), Fisher’s exact test was used in statistical analysis. **(B)** Venn diagrams show the intersections among EE (Evening Element genes), CBS (CCA1 Binding Site genes), SIG (Senescence Induced Genes), SRG (Senescence Repressed Genes), PRS (Positive Regulate natural Senescence genes), and NRS (Negative Regulate natural Senescence genes). The genes belong to indicated groups are listed in the table below. **(C)** Rhythmic expressions of *CCA1*, *ORE1*, *GLK2*, and *CAB* in the 5th and 6th true leave from 12-h light/12-h dark and then transferred to constant light. Shaded areas indicate subjective night periods. The leaves were harvested at indicated time points. Three biological replicates were performed. *ACTIN2* is an internal control for quantitative real-time PCR.

Two *cis*-elements related to circadian clocks have been well-documented: CCA1-Binding Site (CBS, AAMAATCT) and Evening Element (EE, AAAATATCT) (Harmer and Kay, 2005). To examine whether circadian regulated *cis*-elements are enriched in the promoter regions of senescence-associated genes (SAGs), we used the Patmatch tool on the TAIR website^2^ to search for CBS or EE in gene promoter regions (1000 bp sequence upstream of translation start site). According to the Tair 10 genome background, 2330 genes (6.93%) with one or more EEs and 9134 genes (27.18%) with one or more CBSs were identified among 33,602 gene promoter regions. When we examined 1961 senescence induced genes (SIGs) and 1554 senescence repressed genes (SRGs) during natural senescence (van der Graaff, 2006), we found that 30.04% (589/1961) of the SIGs and 28.44% (442/1554) of the SRGs had one or more CBSs, whereas 8.77% (172/1961) of the SIGs and 9.07% (141/1554) of the SRGs had one or more EEs in their promoters. Genes with promoter bearing either CBSs or EEs showed significant enrichment in SIGs relative to the whole genome background, and those bearing EE showed enrichment in SRGs (Fisher’s exact test, **Figure 2A**), indicating that senescence associated genes might be widely under the circadian regulation.

We obtained 589+442 CBS-containing SIG/SRG genes and 172+141 EE-containing SIG/SRG genes. We further looked for the overlap between these candidates with 91 Positive Regulate natural Senescence genes (PRS) and 92 Negative Regulate natural Senescence genes (NRS) (Li et al., 2014) (**Figure 2B** and Supplementary Table 1). The overlapped genes could be the putative senescence associated targets of CCA1. Taking advantage of Diurnal database^3^, the diurnal transcriptional expression of some candidates were displayed in Supplementary Figure 3.

The positive senescence regulators *ORE1* (Kim et al., 2009; Qiu et al., 2015) and chloroplast activity maintainer gene *GLK2* (Waters et al., 2009; Rauf et al., 2013) appeared in the circadian regulated gene list and their expressional patterns are rhythmic (**Figure 2B** and Supplementary Figure 3). Then we conducted a 48-h time-course experiment to test the expression of *CCA1, ORE1*, *GLK2*, and *CAB* in 4-week old Col-0 plants grown under 12L/12D and following by constant light. The expression levels of *ORE1* and *GLK2* exhibited a diurnal rhythm, indicating that CCA1 might regulate the expression of these two genes (**Figure 2C**).

### CCA1 Negatively Regulates Leaf Senescence Through Repressing *ORE1* and Activating *GLK2*

**FIGURE 3:**
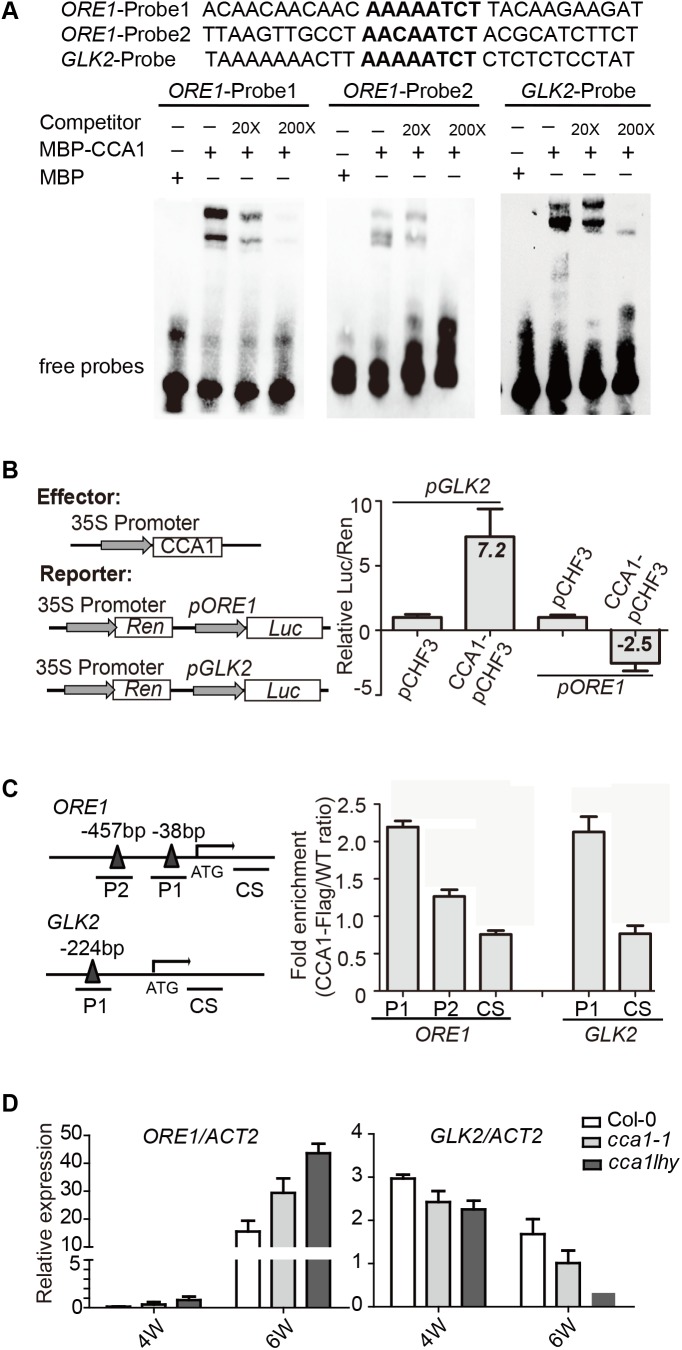
CCA1 binds to the promoters and regulates gene expression of *ORE1* and *GLK2*. **(A)** CCA1 protein physically interacts with *GLK2/ORE1*’s promoters in EMSA assay. MBP protein was used as a negative control. The probes are listed on the top. **(B)** Effects of CCA1 on the activity of *GLK2*/*ORE1* promoters in tobacco leaves. Effector construct contains CCA1 driven by CaMV 35S promoter. Reporter construct contains the Luc reporter gene driven by the promoter of *GLK2/ORE1*. Effects of CCA1 on *GLK2/ORE1*’s promoter were expressed as a ratio of Luc to Ren. Relative Luc/Ren was normalized to control (pCHF3). **(C)** Binding of CCA1 with the promoter of *GLK2*/*ORE1 in vivo*. ChIP assays were quantified by real-time PCR after normalizing with wild type samples. The fragments of GLK2/ORE1 coding sequences (CS) were used as negative control. Bars represent SEM, two independent experiments. Black triangles represent CBS *cis*-element. **(D)** Relative expressions of *GLK2*/*ORE1* in 4- and 6-week-old Col-0, *cca1-1*, and *cca1lhy* double mutants. Three biological replicates were performed. *ACTIN2* is an internal control for quantitative real-time PCR.

To address the direct or indirect regulation of CCA1 on *ORE1* and *GLK2*, we searched the CBS motif in promoter region of *ORE1* and *GLK2*. There are two putative CBSs [-38 to -44, -457 to -465 bp upstream of TSS (translation start site)] in *ORE1’s* promoter and one CBS at 224 bp from the TSS in *GLK2’s* promoter. Electrophoretic mobility shift assay (EMSA) showed that an MBP-CCA1 fusion protein purified from *E. coli* binds to those CBSs in the promoters of both *ORE1* and *GLK2* (**Figure 3A**). The binding of CCA1 with *ORE1-Probe1* appears to be stronger than that with *ORE1-Probe2*, probably due to 1 bp difference on conserved binding motif. Non-labeled DNA competitor successfully eliminated the binding ability in a dosage-dependent manner, suggesting that the binding of CCA1 to those probes is specific.

By fusing the *ORE1* and *GLK2* promoters with the luciferase gene, their transcriptional activation by overexpressed *CCA1* can be tested in tobacco leaves. We found that CCA1 had a 7.2-fold activation effect on *GLK2*’s promoter and an approximately 2.5-fold inhibitory effect on *ORE1*’s promoter (**Figure 3B**).

To verify whether CCA1 interacts with *ORE1/GLK2*’s promoters *in vivo*, a 4-week-old transgenic line harboring *35S:CCA1-Flag* in the Col-0 background was used for ChIP-qPCR. ChIP assays revealed that CCA1 was able to bind to the promoters of *ORE1* and *GLK2*, including both the P1 and P2 regions, which contain CBS elements (**Figure 3C**).

Moreover, higher expression of *ORE1* and lower expression of *GLK2* were found in 6-week-old *cca1-1* and *cca1lhy* mutants compared to that in Col-0 by qRT-PCR (**Figure 3D**).

These results collectively indicate that CCA1 directly binds to the promoters of *ORE1* and *GLK2* and regulates their expression.

### ORE1 and GLK2 Act Downstream of CCA1

**FIGURE 4:**
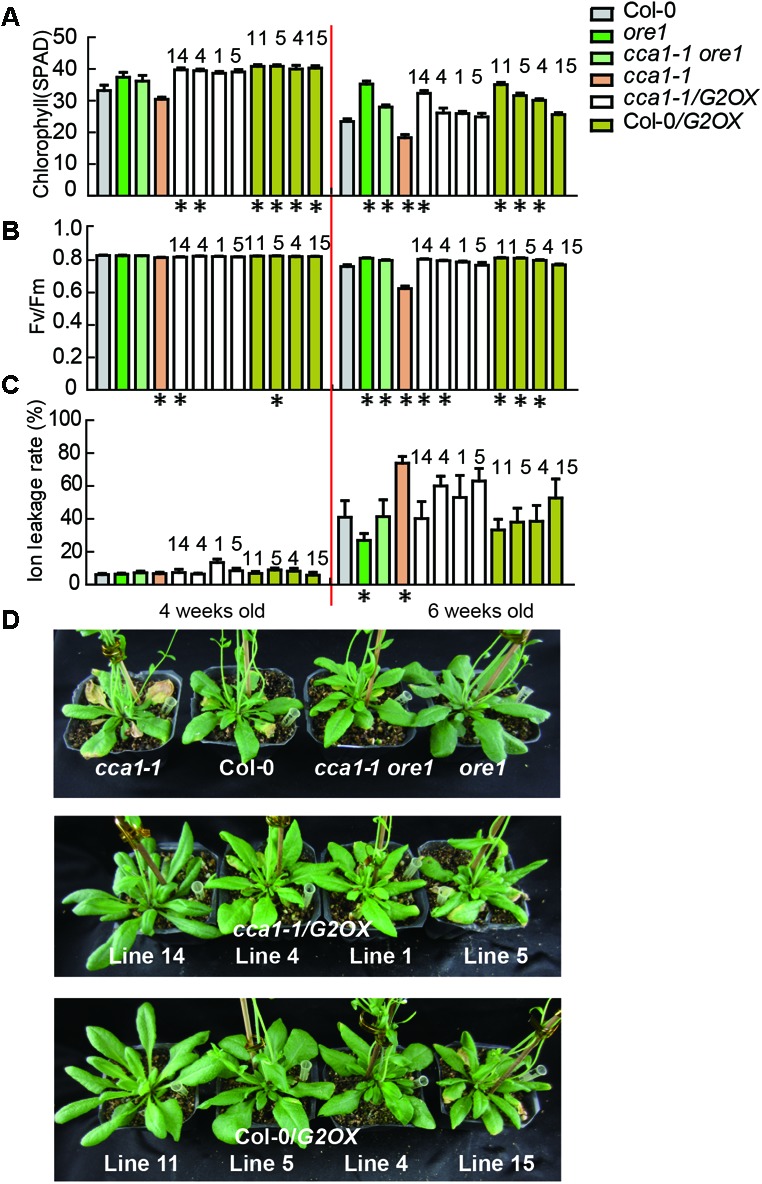
ORE1 and GLK2 act genetically downstream of CCA1. Chlorophyll contents (measured as SPAD) **(A)** Fv/Fm ratios **(B)** and ion leakage rates **(C)** of the 5th and 6th true leaves from different genotypes measured at 3, 4, 5, and 6 weeks under long day after germination. Four independent *GLK2* overexpressed lines in Col-0 (Col-0/G2OX) or *cca1-1* (*cca1-1/*G2OX) were shown. Asterisks indicate where the difference between Col-0 and mutant is statistically significant (^∗^*P* < 0.05). Numbers indicate different independent lines. **(D)** Phenotypes of different genotypes at 6 weeks under long day after germination.

To discover the genetic relationship between CCA1 and its two target genes *ORE1* and *GLK2*, we generated a *cca1-1ore1* double mutant and ectopically expressed *35S:GLK2* in the *cca1-1* background. The expression levels of *CCA1*, *ORE1*, and *GLK2* were confirmed by qRT-PCR and western blotting in the above lines (Supplementary Figure 4). As expected, the *cca1-1ore1* double mutant and *cca1-1/G2OX* (*35S:GLK2/cca1-1*) showed similar senescence process with *ore1* and Col-0*/G2OX* (*35S:GLK2/*Col-0) (16L/8D), respectively. The earlier senescence phenotype of *cca1-1* was reversed by knockdown of *ORE1* and overexpression of *GLK2* based on the measurement of chlorophyll content, Fv/Fm and ion leakage (**Figures 4A–D**). These data demonstrate that CCA1 negatively regulates senescence through repressing the expression of *ORE1* and activating the expression of *GLK2*.

### Age-Declined CCA1 Attenuates the Inhibition of Leaf Senescence

**FIGURE 5:**
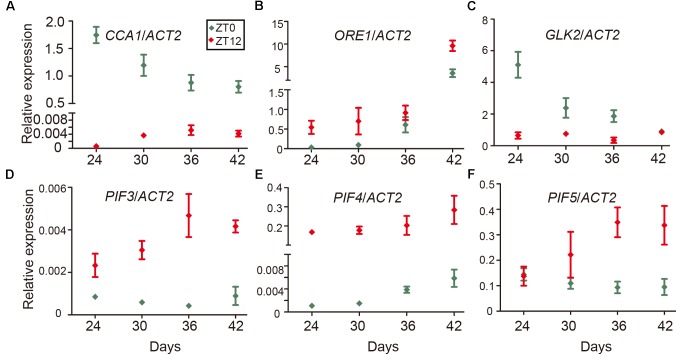
Expression changes of *CCA1*, *ORE1*, *GLK2*, and *PIF*s with aging. Relative expression levels of *CCA1* **(A)**, *ORE1* **(B)**, *GLK2* **(C)**, *PIF3* **(D)**, *PIF4* **(E)**, and *PIF5* **(F)** in the 5th and 6th true leaves of wild type from the indicated days (Y-axis) after germination. Relative expression of genes at ZT0 and ZT12 were measured for each time point. Bars represent the mean with SEM, *n* = 3.

Even as the circadian rhythm efficiently restricts leaf senescence during the juvenile stage, senescence occurs eventually as the leaf ages. One explanation is a reduction of the expression of circadian-related genes during aging. To test this idea, we performed time-course qRT-PCR to monitor the expression of *CCA1* at both dawn and dusk at 24, 30, 36, and 42 days. We collected samples at ZT0 and ZT12 since the mRNA level of *CCA1* peaked at dawn (ZT0) and decreased to a low level at dusk (ZT12) (Wang et al., 2011). Interestingly, although the expression of *CCA1* oscillated between dawn and dusk, the expression and the rhythmically amplitude were significantly decreased with aging (**Figure 5A**). Moreover, besides *CCA1*, the expressions of circadian component *ELF3* and *CHE* were also decreased and the rhythmically amplitudes of *CO* and *JMJD5* dramatically reduced with aging (Supplementary Figure 5). With transcriptional decline of *CCA1*, the expression levels of *ORE1* and *GLK2* followed their regulation by CCA1 (**Figures 5B,C** and Supplementary Figure 5). Further, the expression of *PIF3*, *PIF4* and *PIF5* increased with aging (**Figures 5D–F** and Supplementary Figure 5). Thus, age-declined CCA1 and GLK2 attenuate the inhibition of leaf senescence and accumulated PIFs and ORE1 promote senescence initiation.

## Discussion

Leaf senescence is a complex degenerative process under tight genetic control and also affected by environmental signal. Circadian resonance is likely to provide advantageous adaptation in all kingdoms, including plants. It has been reported that plant circadian clocks are involved in the regulation of photosynthesis, growth, survival, and competitive advantage (Dodd et al., 2005). Whether circadian clocks prevent leaf senescence in plants is not clear. Several senescence-associated genes have been reported to be regulated by circadian clocks. For example, the abundance of NPR1 (Non-expressor of Pathogenesis-Related gene 1) is circadian gated (Zheng et al., 2015; Zhou et al., 2015) and promotes leaf senescence (Morris et al., 2000; Fitzgerald et al., 2004). PIFs, the positive regulators of leaf senescence (Sakuraba et al., 2014; Song et al., 2014), diurnally oscillate at the transcriptional/translational levels (Soy et al., 2016). Circadian evening complex component ELF3 regulates dark-induced leaf senescence (Sakuraba et al., 2014). These findings raised the question of whether the circadian clock regulates plant senescence.

**FIGURE 6:**
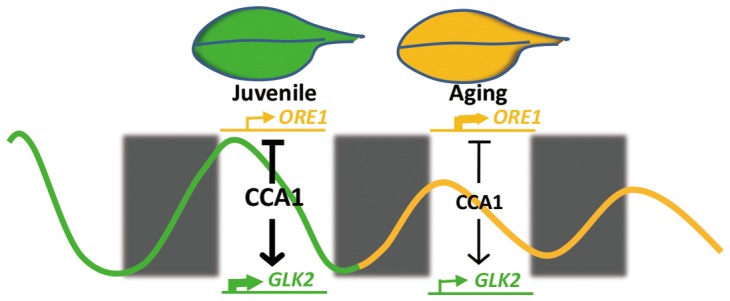
A proposed model illustrating the crosstalk between CCA1 and leaf senescence.

Our current study demonstrates that central circadian clock component CCA1 appears to regulate the expression of *ORE1* and *GLK2* to inhibit leaf senescence at the juvenile stage. With aging, declined CCA1 releases the inhibition and promotes the senescence initiation (**Figure 6**). This proposed mechanistic model is based on several evidences: (i) *cca1* displays the accelerated yellowing phenotype; (ii) *ORE1* and *GLK2* belong to targets of CCA1; (iii) CCA1 directly binds to the promoter of *ORE1* and *GLK2*; (iv) the expression of *ORE1* and *GLK2* is altered in *cca1*; (v) knocking down the expression of *ORE1* and overexpressing of *GLK2* rescued the earlier senescence phenotype of *cca1*; (vi) the transcriptional level of *CCA1* decreased with aging.

As a central circadian rhythm regulator, CCA1 occupied more than 1000 genomic regions identified in the seedling stage according to ChIP sequencing data (Nagel et al., 2015). Among the targets of CCA1, although many of the target genes are repressed by CCA1, some of them are still activated, such as *LHCB1.1* (*CAB2*) (Wang et al., 1997; Wang and Tobin, 1998). The flanking regions of CBS or EE and different interacting partners of CCA1 might modulate the expression of target genes and lead to the different action of CCA1 (Harmer and Kay, 2005). The confirmation of target genes of CCA1 emphasizes the broad role of the plant clock in regulating multiple biological functions. Based on our bioinformatics comparative analysis, *ORE1* and *GLK2* were identified and experimentally confirmed in ChIP-PCR assay using pre-senescence leaves. However, they are not shown in ChIP-Sequencing dataset using young seedlings, probably because co-partners of CCA1 are different during distinct developmental stages. Several interacting partners of CCA1 in early developmental stage have been published (Sugano et al., 1998; Andronis et al., 2008; Lau et al., 2011). Future work to identify CCA1 co-partners during senescence stage will be desired. Besides *ORE1* and *GLK2*, more targets of CCA1 probably also contribute to leaf senescence, such as photosynthesis related genes including *PORB* (Ni et al., 2009), reactive oxygen species (ROS) homeostasis related genes (Lai et al., 2012).

Interestingly, CCA1 positively regulates *GLK2* and negatively regulates *ORE1* expression, which was reminiscent of a similar model that stress related senescence regulator ATAF1 executes its physiological role by simultaneously affecting both *GLK1* and *ORE1* (Garapati et al., 2015). Decreased expression of *GLKs* and enhanced accumulation of *ORE1* constitutes a molecular transition mechanism from growth to senescence. These two key molecules of leaf senescence were regulated not only by CCA1, also by several modulators, such as EIN3 (Qiu et al., 2015), PIF4/5 (Sakuraba et al., 2014; Song et al., 2014). With aging, the dramatically transcriptional changes of these two genes were co-regulated by multiple signal pathways in senescence initiation.

In addition to the role of CCA1 on downstream gene regulation, the misalignment of the internal circadian clock and the external influence of *zeitgebers* in *cca1* leads to complicated effects on plant metabolism that probably also contribute to reduced longevity in our detected condition. To assess the effect of circadian period on leaf senescence, we conducted a preliminary experiment to detect the senescence phenotype of plants grown in two distinct photoperiods (Photoperiod 1: 16 h light and 8 h dark; Photoperiod 2: 8 h light and 4 h dark). Plants grown in 16L/8D and 8L/4D showed similar chlorophyll content and Fv/Fm ratio at 4 weeks after sowing; however, plants in 8L/4D showed a more severe leaf yellowing phenotype relative to plants in 16L/8D at 6 weeks (Supplementary Figure 6), which indicates that accelerated circadian rhythm promotes leaf senescence in *Arabidopsis*. Besides the effect of photoperiod on leaf senescence, future work will be also required to figure out whether the circadian clock components regulate leaf senescence though modulating the hormone responses.

Taken together, we provided insights to how a circadian clock component regulates leaf senescence and how the expression of *CCA1* is changed during aging. Our work suggests that circadian system acts as a timekeeper of natural senescence and enhances survival by timing initiation of leaf senescence.

## Author Contributions

YS, YJ, BK, and LL designed the research; YS and YJ performed the experiments; YS, YJ, and LL analyzed the data; LL wrote the manuscript. All authors read and approved the final manuscript.

## Conflict of Interest Statement

The authors declare that the research was conducted in the absence of any commercial or financial relationships that could be construed as a potential conflict of interest.

## Footnotes

1 psd.cbi.pku.edu.cn/

2 www.arabidopsis.org

3 http://diurnal.mocklerlab.org/
