# Supplementary material for: CIRCADIAN CLOCK-ASSOCIATED 1 Inhibits Leaf Senescence in Arabidopsis
Source: Front Plant Sci. 2018 Mar 6;9:280. doi: 10.3389/fpls.2018.00280 (PMC5845730; doi:10.3389/fpls.2018.00280)
Supplement: Supplementary file 2 [file Table_2.PDF]

Supplementary Table 2. Primers used in this study.

For qPCR experiment

CCA1-S	GAGGCTTTATGGTAGAGCATGGCA
CCA1-AS	TCAGCCTCTTTCTCTACCTTGGAGA
ORE1-S	CTGTTTTACTCGGATCCTCTGTTT
ORE1-AS	AAACGCAATCCAATTCTTCTGTAC
GLK2-CS-S	GGTCACATCGGAAACATCTACT
GLK2-CS-AS	TCTTCCCTCCTCCTCCTACTC
PIF3-S	CTCGTTGACAGTAACAGGAGAC
PIF3-AS	GAATCTGCTCAAGACAGGAAC
PIF4-S	CGACTCAGCCGATGGAGATGTT
PIF4-AS	GTTGTTGACTTTGCTGTCCCGC
PIF5-S	GCGGGAAATCAGACCGTGCAACAA
PIF5-AS	CGCCGGAGATCCAAATCCCAACAT
ACT2-S	CGCTCTTTCTTTCCAAGCTC
ACT2-AS	AACAGCCCTGGGAGCATC

EMSA probes

ORE1-Probe1-S	ACAACAACAACAAAAATCTTACAAGAAGAT
ORE1-Probe2-S	AATGTGATAAGAACAATCTTATCACATAAA
GLK2-Probe-S	TAAAAAAACTTAAAAATCTCTCTCTCCTAT
ORE1-1-S	ACAACAACAACAAAAATCTTACAAGAAGAT
ORE1-1-AS	ATCTTCTTGTAAGATTTTTGTTGTTGTTGT
ORE1-2-S	AATGTGATAAGAACAATCTTATCACATAAA
ORE1-2-AS	TTTATGTGATAAGATTGTTCTTATCACATT
GLK2-S	TAAAAAAACTTAAAAATCTCTCTCTCCTAT
GLK2-AS	ATAGGAGAGAGAGATTTTTAAGTTTTTTTA

For Plasmid Construction

PromGLK2-0800	ATA GTCGAC AGAAAAACCAAGGAGAAAACAAA
PromGLK2-0800	ATA GGATCC CGGAATCGTAAAAAAATGAAAA
PromORE1-0800	ATA CTCGAG TTCTTTTGAATGTATGCATCTAAC
PromORE1-0800	ATA GCGGCCGC TTTATCCTAATAGGGTTTCTAAAAA
CCA1-S-Pchf3	ATA GGTACC ATGGAGACAAATTCGTCTGGAG
CCA1-AS-Pchf3	ATA GTCGAC TCATGTGGAAGCTTGAGTTTCC
GLK2-CDS-S	ATA GGTACC ATGTTAACTGTTTCTCCGGCTCC
GLK2-CDS-AS	ATA GTCGAC AGGAAGAGGAGGAACATTAGAAA

For ChIP-qPCR

PGLK2-S	TCCAAAATGTAAAAAAAAATCACT
PGLK2-AS	CTTGGTTGTCCCAAAAATAAAG
GLK2-CS-S	GGTCACATCGGAAACATCTACT

GLK2-CS-AS	TCTTCCCTCCTCCTCCTACTC
PORE1-BS1-S	TGCTCAAACACTTTCCTCTCTC
PORE1-BS1-AS	CCTCGTAATCCATTTTATCCTA
PORE1-BS2-S	TGAAAACGATATTTGAGAATCAC
PORE1-BS2-AS	AGAGAAAAGAAGAAGATGCGTAG
ORE1-CS-S	CCCCAAACAGCTAAGGTAATAA
ORE1-CS-AS	TACATTCACAAACAACCAAACA
	**For Genotyping**
CCA1-1-LP	TGAGATTTCTCCATTTCCGTAGCTTCTGG
CCA1-1-RP	ATCCGTTTGGGATCTTTCTGTTCCACATG
CCA1-1-LB	GATGCACTCGAAATCAGCCAATTTTAGAC
ORE1-LP	CGATCTTAGGGTTACGTTGGG
ORE1-RP	ATCTTCCCCAAACAGCTAAGG
LB1.3	ATTTTGCCGATTTCGGAAC
