# Supplementary figures and images for: CIRCADIAN CLOCK-ASSOCIATED 1 Inhibits Leaf Senescence in Arabidopsis
Source: Front Plant Sci. 2018 Mar 6;9:280. doi: 10.3389/fpls.2018.00280 (PMC5845730; doi:10.3389/fpls.2018.00280)

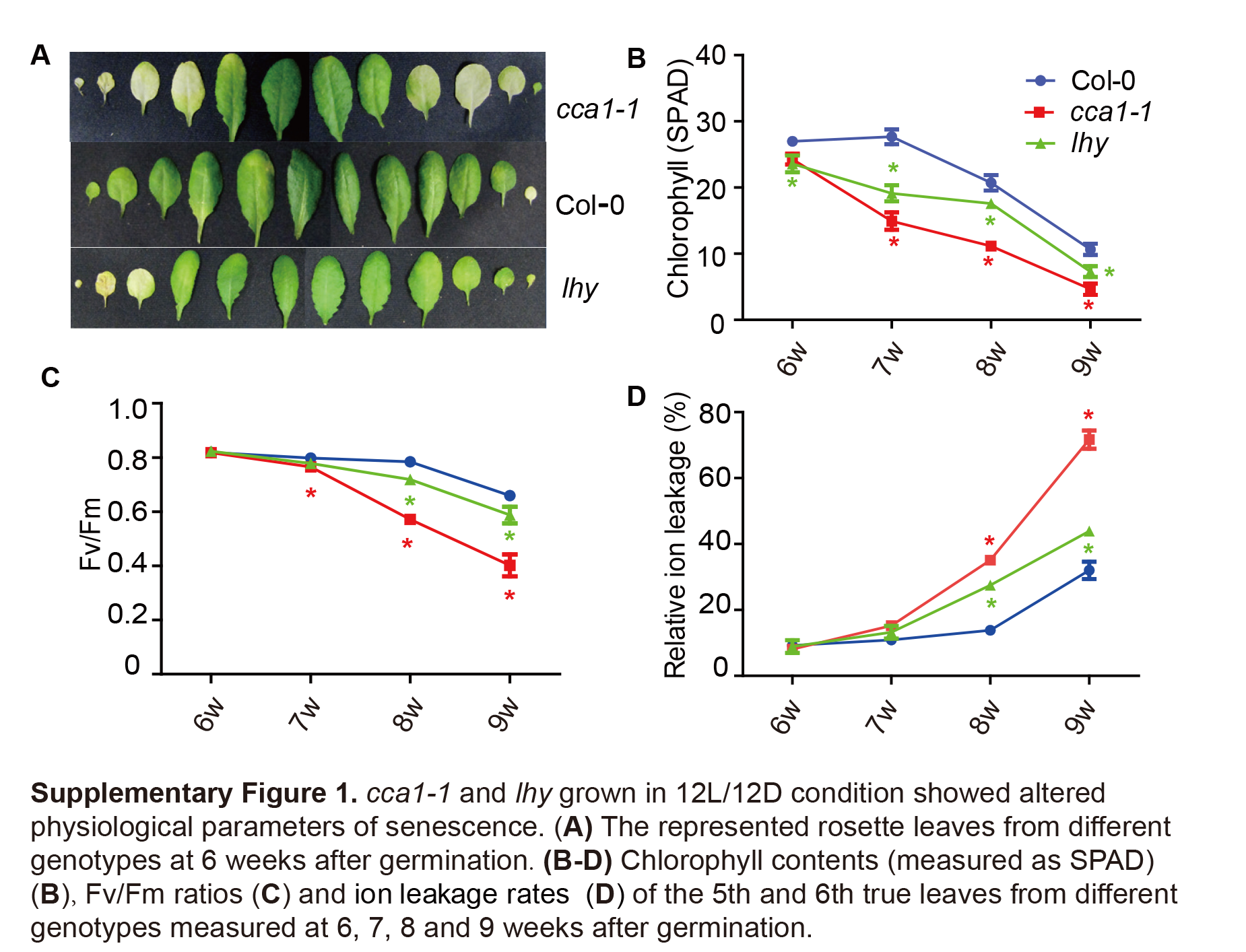

Supplement: Supplementary file 3 [file Image_1.TIF]

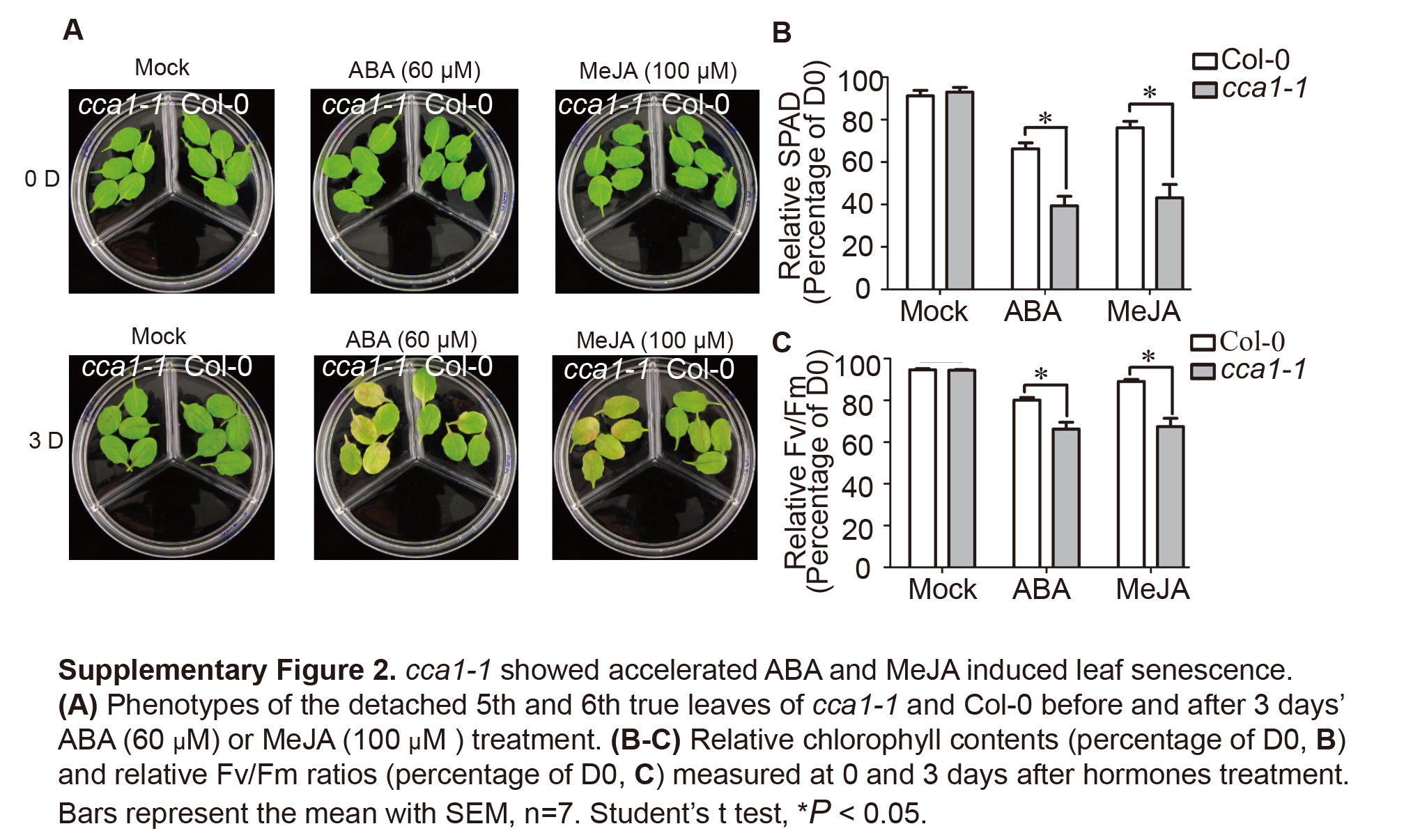

Supplement: Supplementary file 4 [file Image_2.TIF]

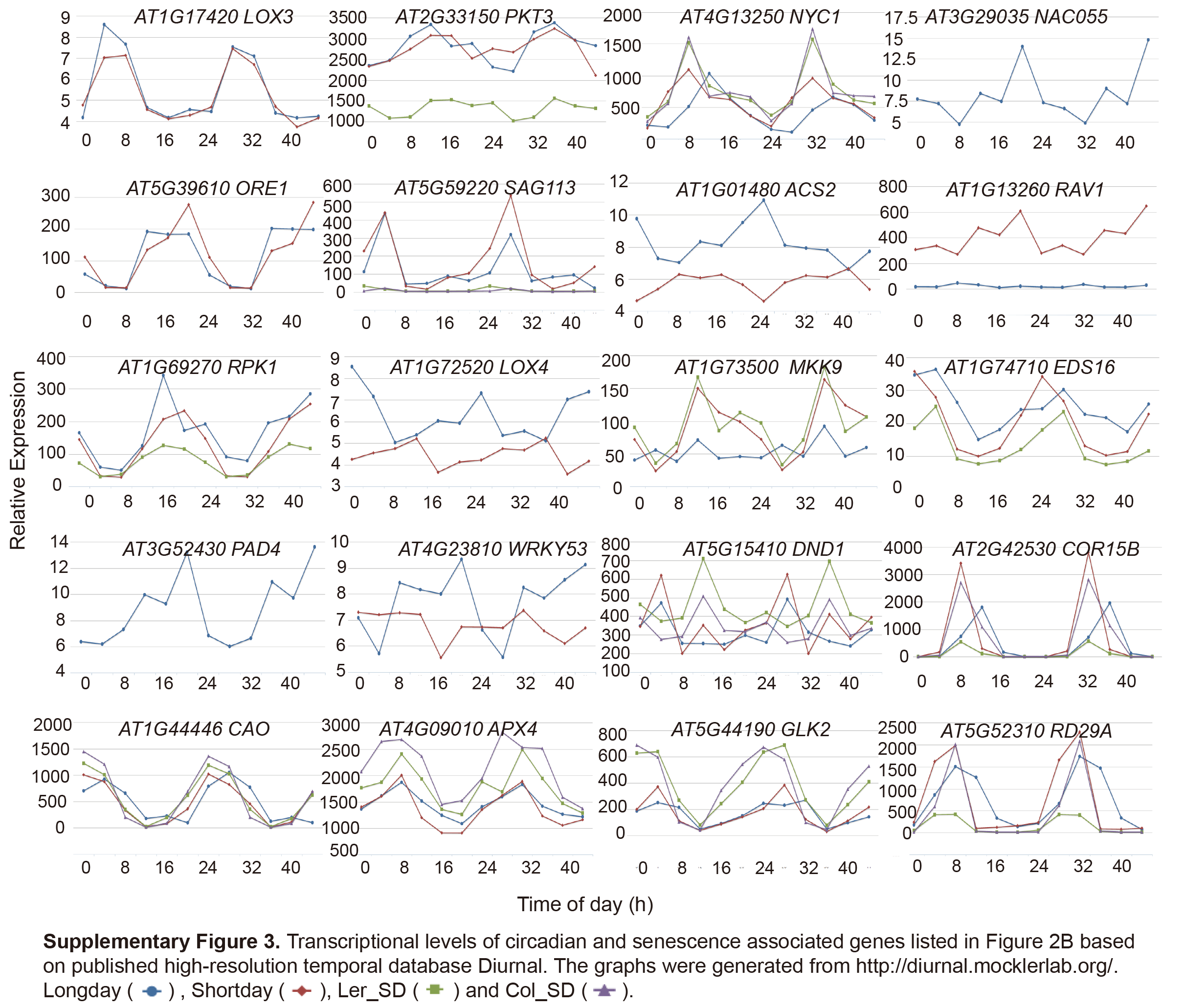

Supplement: Supplementary file 5 [file Image_3.TIF]

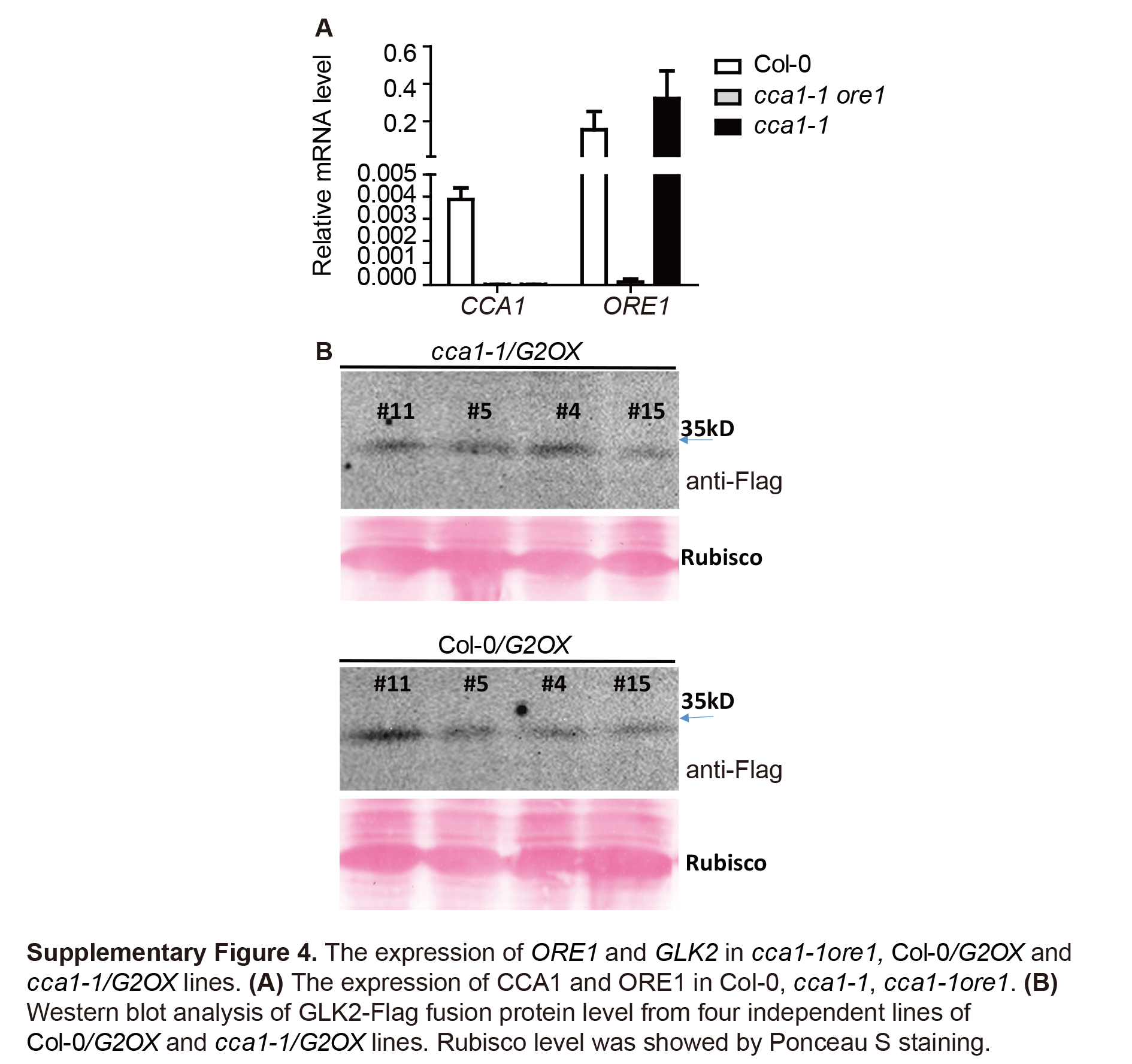

Supplement: Supplementary file 6 [file Image_4.TIF]

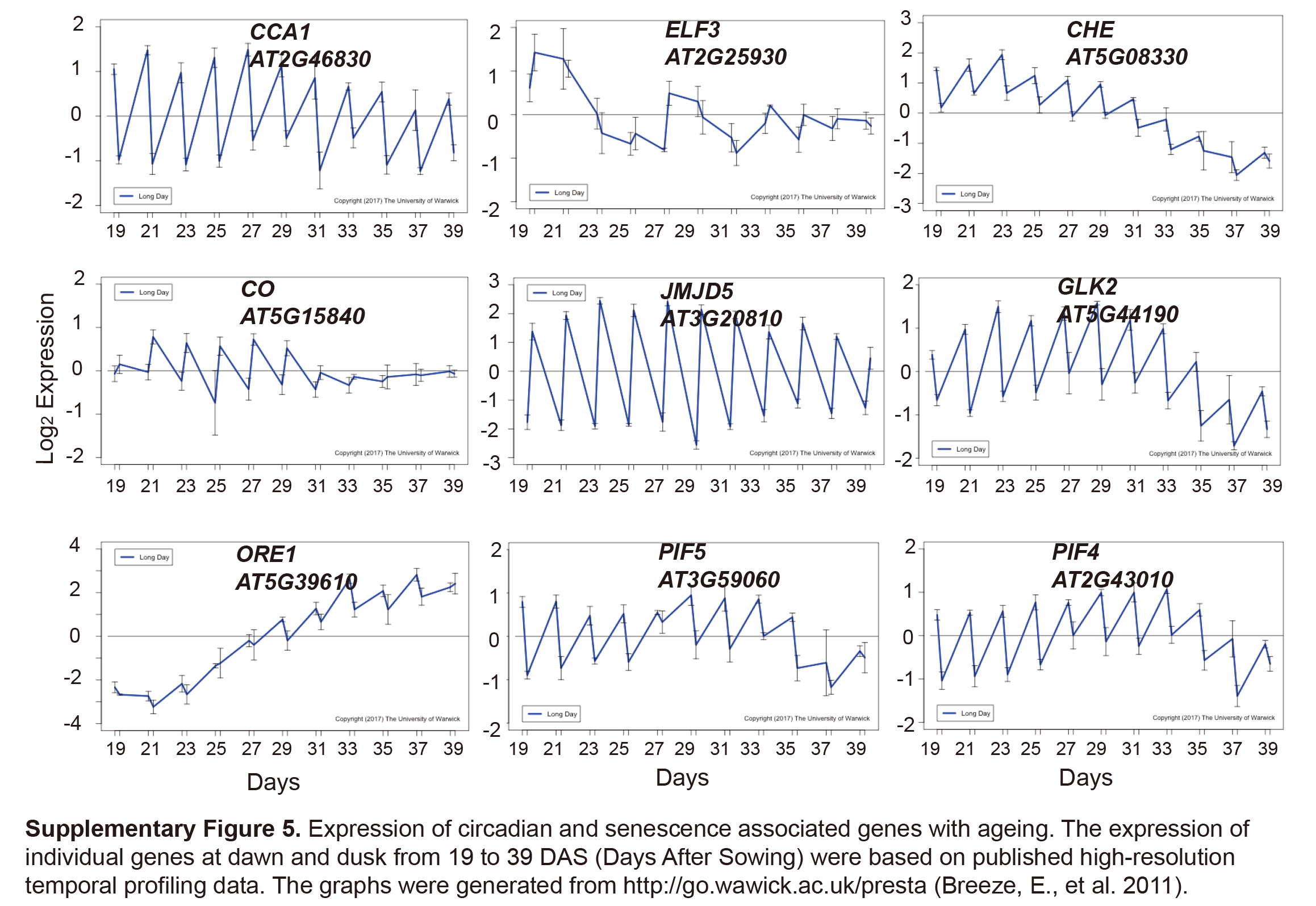

Supplement: Supplementary file 7 [file Image_5.TIF]

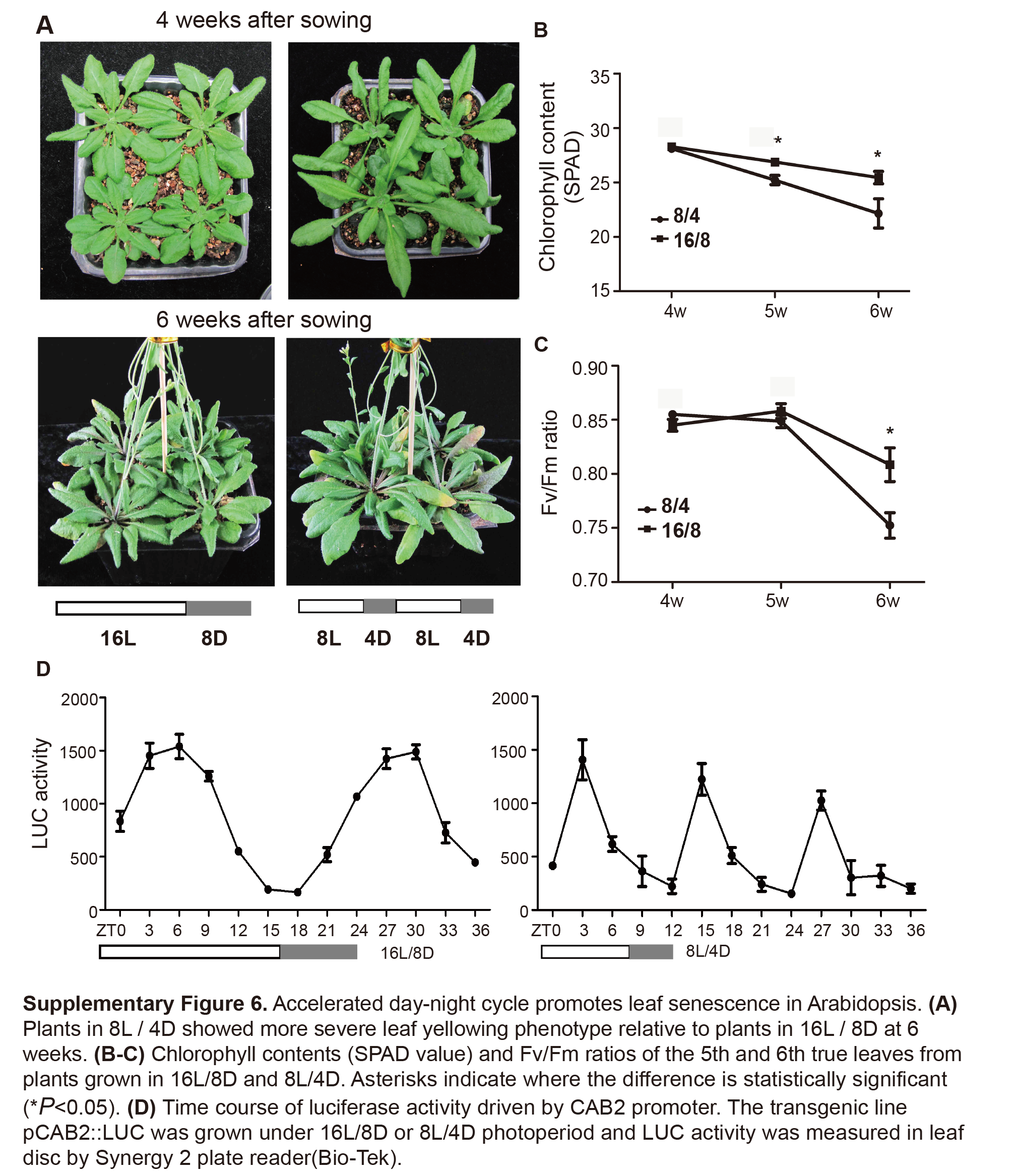

Supplement: Supplementary file 8 [file Image_6.TIF]
